# Physical Exercise and Health: A Focus on Its Protective Role in Neurodegenerative Diseases

**DOI:** 10.3390/jfmk7020038

**Published:** 2022-04-29

**Authors:** Roberto Bonanni, Ida Cariati, Umberto Tarantino, Giovanna D’Arcangelo, Virginia Tancredi

**Affiliations:** 1 Department of Clinical Sciences and Translational Medicine, “Tor Vergata” University of Rome, 00133 Rome, Italy; roberto.bonanni1288@gmail.com (R.B.); umberto.tarantino@uniroma2.it (U.T.); 2 Department of Orthopaedics and Traumatology, “Policlinico Tor Vergata” Foundation, 00133 Rome, Italy; 3 Centre of Space Bio-Medicine, “Tor Vergata” University of Rome, 00133 Rome, Italy; giovanna.darcangelo@uniroma2.it (G.D.); tancredi@uniroma2.it (V.T.); 4 Department of Systems Medicine, “Tor Vergata” University of Rome, 00133 Rome, Italy

**Keywords:** physical exercise, brain health, synaptic plasticity, neurotrophins, neuroprotection, neurodegeneration, preventive strategy

## Abstract

Scientific evidence has demonstrated the power of physical exercise in the prevention and treatment of numerous chronic and/or age-related diseases, such as musculoskeletal, metabolic, and cardiovascular disorders. In addition, regular exercise is known to play a key role in the context of neurodegenerative diseases, as it helps to reduce the risk of their onset and counteracts their progression. However, the underlying molecular mechanisms have not yet been fully elucidated. In this regard, neurotrophins, such as brain-derived neurotrophic factor (BDNF), nerve growth factor (NGF), glia cell line-derived neurotrophic factor (GDNF), neurotrophin-3 (NT-3), and neurotrophin-4 (NT-4), have been suggested as key mediators of brain health benefits, as they are involved in neurogenesis, neuronal survival, and synaptic plasticity. The production of these neurotrophic factors, known to be increased by physical exercise, is downregulated in neurodegenerative disorders, suggesting their fundamental importance in maintaining brain health. However, the mechanism by which physical exercise promotes the production of neurotrophins remains to be understood, posing limits on their use for the development of potential therapeutic strategies for the treatment of neurodegenerative diseases. In this literature review, we analyzed the most recent evidence regarding the relationship between physical exercise, neurotrophins, and brain health, providing an overview of their involvement in the onset and progression of neurodegeneration.

## 1. Introduction

Physical exercise is a non-pharmacological strategy for various disease conditions, as well as for maintaining general health [1]. Numerous studies have shown that regular moderate-intensity physical exercise can bring enormous benefits to the entire body, reversing at least some of the deleterious effects of a sedentary lifestyle and providing valuable support for longevity [2,3,4].

The main benefits have been observed at the metabolic and cardiorespiratory level, as physical exercise is known to promote better control of blood pressure and circulating cholesterol levels, a better ability of tissues, especially muscle tissue, to take up oxygen from the blood and remove carbon dioxide as a waste product, and a decrease in fat deposits, especially visceral fat, which is mainly responsible for the onset of chronic diseases, such as metabolic syndrome, obesity, inflammation, and type 2 diabetes mellitus [5,6,7,8,9].

Given the strong association of pathological conditions, such as hypertension with alterations in the blood-brain barrier and brain dysfunction, physical exercise may also have beneficial effects on cerebrovascular and cognitive functions, helping to delay brain aging and the onset of degenerative conditions, such as Alzheimer’s disease (AD) and Parkinson’s disease (PD) [10,11]. In addition to improving cognitive and memory processes by regulating the expression of growth factors and neurotrophins, physical exercise also has a psychosomatic effect by promoting the release of endorphins, a particular group of chemicals produced by the brain that have a powerful analgesic effect [12].

Finally, anti-inflammatory effects of physical exercise have been reported, which may vary depending on the training protocol used. While a lifestyle with regular exercise, compared to a sedentary lifestyle, can increase immune competence and reduce the risk of infection, too intense and frequent training programs can have negative effects on health [13,14].

In addition to being an effective preventive strategy, proper physical activity has other benefits for the body, such as increasing muscle strength and elasticity, helping to increase joint mobility, promoting flexibility, making movements more fluid, improving coordination and balance, and reducing the risk of injuries and falls, especially for the elderly [15].

Finally, adequate exercise can influence the metabolic health of an individual during embryonic development [16]. Several clinical studies have shown that a mother’s physical exercise during pregnancy not only improves brain maturation in newborns and, thereafter, childhood language development, but also influences an offspring’s susceptibility to disease in adulthood through fetal metabolic programming [17,18]. In this regard, Klein et al. have recently shown that maternal exercise during pregnancy has long-lasting metabolic effects on the offspring’s brain, particularly on mitochondrial function. Specifically, they observed a neuroprotective role against β-amyloid (Aβ) neurotoxicity and cognitive impairment in adulthood, and proposed maternal exercise as a promising strategy to delay or even prevent the development of AD [19].

Thus, adopting an active lifestyle is undoubtedly one of the best strategies for preventing degenerative diseases, maintaining general health, and improving quality of life. In this regard, the aim of our review was to investigate the impact of physical exercise on brain health, summarizing the current knowledge on the underlying cellular and molecular processes and highlighting how physical exercise may represent an effective alternative strategy for preventing and/or counteracting neurodegeneration related to aging and/or a sedentary lifestyle.

## 2. Literature Search Strategy

For this narrative review, 114 papers were selected from the Medline bibliographic database, published between 1945 (starting date) and 2022. The selected papers included studies of the impact of physical exercise on cellular and molecular processes underlying brain health and neurodegeneration. The search strategy was based on the use and/or combination of the following keywords: “physical exercise”; “physical activity”; “brain health”; “cellular mechanisms”; “molecular mechanisms”; “synaptic plasticity”; “neurotrophins”; “neurodegeneration”; and “neuroprotection”. The search process was performed on a worldwide basis, without excluding specific geographic areas or different ethnic groups. Language filters and species filters were applied to the results list to eliminate non-English articles.

## 3. Effects of Physical Exercise on Brain Health

In recent years, a growing body of scientific evidence has demonstrated the broad effects of physical exercise on general brain health in animals and humans, especially for learning and memory processes, protection from neurodegeneration, and alleviation of depression [20,21]. Several mechanisms have been proposed to explain the influence of physical exercise on brain function, particularly an exercise-dependent central and peripheral regulation of several growth factors involved in neurogenesis, metabolism, and vascular function. Accordingly, these factors may be responsible for the structural and functional brain changes underlying synaptic plasticity [22].

### 3.1. Physical Exercise Improves Synaptic Plasticity

Synaptic plasticity, a fundamental property of neurons, consists of activity-dependent changes in the strength and efficiency of synaptic transmission of pre-existing neuronal connections [23]. These changes, which can last from a few milliseconds to hours or even days, are primarily responsible for learning and memory processes, as well as for the development of the brain’s response to injury [24]. Synaptic plasticity can result in a persistent increase in synaptic strength, termed long-term potentiation (LTP), or a sustained reduction in synaptic strength, known as long-term depression (LTD) [25].

Over the last 40 years, the phenomena of synaptic plasticity have been studied and described in detail. However, the underlying molecular mechanisms are extremely complex and the ways in which synaptic plasticity is induced in vivo or during learning, or altered in neurodegenerative disorders, have yet to be fully explored [26].

Physical exercise is certainly one of the events that can induce synaptic plasticity. Indeed, numerous studies on animal models have shown how regular exercise can improve learning and memory processes and counteract age-related cognitive decline [27,28]. In this regard, we recently evaluated the effects of an aerobic training protocol, administered three times a week for twelve consecutive weeks, on the synaptic plasticity of 4-month-old mice through electrophysiological recordings of hippocampal slices and ultrastructural analysis of the hippocampus. In agreement with previous data [29], aerobic training not only promoted an increase in synaptic plasticity compared to control mice, but also improved hippocampal ultrastructure, as demonstrated by the complete structural conservation of mitochondria, neurofilaments, and neurotubules, highlighting the effectiveness of aerobic training in improving learning and memory processes [30].

The effects of physical exercise on brain health are most evident in mouse models of aging, in which a recovery in cognitive function has been observed in active animals compared to sedentary animals. Recently, Tsai et al. subjected young, adult, and old mice to treadmill training and found a significant neuroprotective effect of exercise on age-related brain changes, such as reduced dendritic length and branching and a decreased number of spines in CA1 neurons [31]. Specifically, moderate-intensity treadmill running exercise for 6 weeks was reported to increase hippocampal synaptic plasticity and to preserve dendritic complexity in all three experimental groups, as well as restoring learning and memory in old mice [31]. Similarly, Li and colleagues assessed the effects of treadmill running for 6 days a week on cognitive function in mouse models of aging, observing an improvement in spatial memory performance and a statistically significant reduction in pro-apoptotic markers in the hippocampus [32]. Taken together, these observations suggest that aerobic exercise plays a key role both in inducing the neuroplasticity involved in learning and memory and in preserving learning and memory during aging by counteracting neuronal death and cognitive decline in later life.

Notably, a significant improvement in synaptic plasticity was also observed after exposure to whole body vibration (WBV) protocols, appropriately designed in terms of vibration frequency, vibration exposure time, and recovery time between training sessions. In this regard, we recently exposed 4-month-old mice to three different WBV protocols and, after 36 training sessions, assessed hippocampal synaptic plasticity using electrophysiological recordings to determine which protocol was most effective in improving learning and memory [33]. The WBV protocol, characterized by a reduced vibration exposure time and a longer recovery period between two contiguous sessions, was observed to promote a significantly better hippocampal response than control animals. Surprisingly, the same WBV protocol administered to a group of 24-month-old animals resulted not only in a marked improvement in synaptic plasticity, but also in the disappearance of signs related to cognitive decline that characterized sedentary old mice [33]. Finally, beneficial effects of WBV training have also been observed in middle-aged mice, including an improvement in synaptic plasticity along with a significant increase in muscle fiber diameter and complete preservation of cellular ultrastructure, suggesting vibratory training as a valid strategy for delaying the onset of symptoms related to a sedentary lifestyle [34].

The beneficial effects of exercise have also been observed in humans, particularly in elderly populations, as regular physical activity has been correlated with improved memory processes and executive function in the brain, less age-related cognitive decline, and greater protection from age-related atrophy in brain areas crucial for higher cognitive processes [35]. Intervention studies in the elderly have demonstrated the effects of various types of exercise, including endurance exercises, balance exercises, and gait retraining and walking, on brain structure, function, and connectivity [36]. In particular, aerobic training is known to increase the volume of grey and white matter in the prefrontal cortex of the elderly, as well as increasing the volumes of the hippocampus and medial temporal lobe, improving spatial memory and reducing the risk of cognitive impairment [37,38]. In this regard, in a randomized controlled trial with 120 elderly people, Erickson et al. showed that hippocampal volume loss in late adulthood is not inevitable, as a 1-year aerobic exercise intervention was effective in increasing hippocampal volume by 2% and compensating for the deterioration associated with aging [39]. Finally, new evidence suggests that the combination of exercise and cognitive testing may increase the potential of prevention and treatment programs to alleviate cognitive decline, confirming the key role of exercise for longevity and more gradual aging [40,41].

### 3.2. Physical Exercise Improves Neurotrophins Production

**Figure 1:**
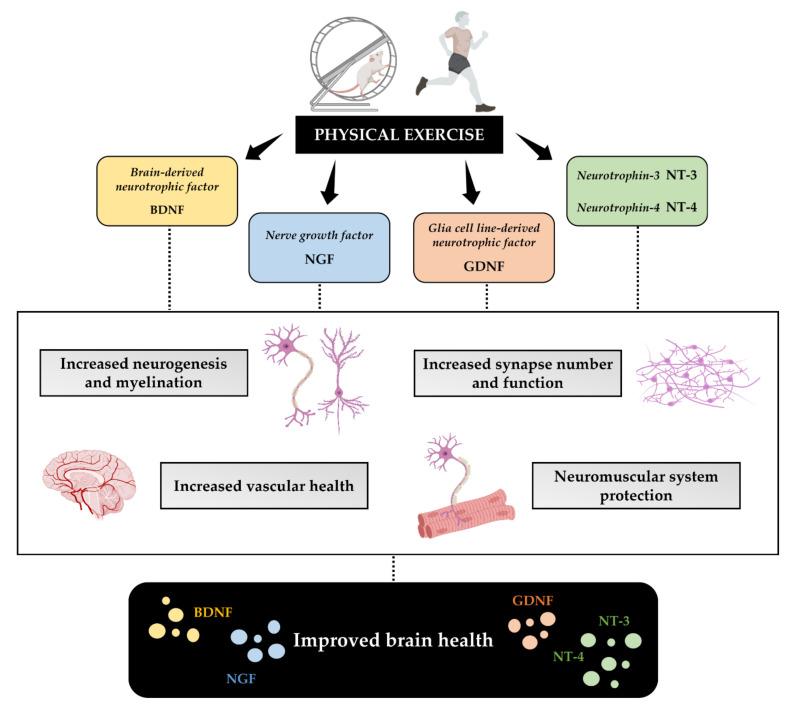
**Physical exercise promotes brain health through the production of neurotrophins.** Neurotrophins are growth factors with a protective role in the central nervous system. Among the most studied are brain-derived neurotrophic factor (BDNF), nerve growth factor (NGF), glial cell line-derived neurotrophic factor (GDNF), neurotrophin-3 (NT-3), and neurotrophin-4 (NT-4), which are released following exercise and promote increased neurogenesis, myelination, and synapse number and function, improve vascular health in the brain, and provide protection to the neuromuscular system.

Among the mediators responsible for the positive impact of physical exercise on brain health, neurotrophins are undoubtedly the leading candidates, as they are involved in numerous events that support structural and functional plasticity in the brain [42]. They are a heterogeneous and pleiotropic group of growth factors involved in a wide number of biological functions, such as the growth and differentiation of new neurons and synapses, the development of axonal and dendritic growth, synaptic plasticity, and neuronal survival [43]. Neurotrophins are important regulators of adult neurogenesis, a phenomenon that involves the formation of new neurons throughout life and occurs in specific brain niches. Therefore, through the production of neurotrophins, physical exercise could promote the preservation of cognitive functions and stimulate neurogenesis, counteracting age-related cognitive decline [44]. Among the most studied neurotrophins for which a neuroprotective role has been suggested, we focused our attention on brain-derived neurotrophic factor (BDNF), nerve growth factor (NGF), glial cell line-derived neurotrophic factor (GDNF), neurotrophin-3 (NT-3), and neurotrophin-4 (NT-4), as shown in Figure 1.

#### 3.2.1. BDNF

BDNF is an essential neurotrophin that controls cognition, neuroplasticity, and angiogenesis, biological activities crucial in the development of learning and memory [45]. Numerous studies have reported that acute and chronic aerobic exercise increases circulating levels of BDNF in a manner dependent on the duration and intensity of exercise [46,47]. In particular, Azevedo et al. recently conducted a systematic review to assess the effects of exercise on circulating BDNF levels in adolescents, finding that adopting an aerobic exercise program was correlated with improvements in BDNF levels. Interestingly, the greatest effects were found in groups of adolescents undergoing moderate- or high-intensity exercise, confirming the dose-response effect that training might exert on neurotrophin production [48]. Similarly, Coelho et al. observed that aerobic exercise, mainly of moderate intensity, significantly increases circulating levels of BDNF in elderly individuals, confirming the ability of aerobic exercise to modulate BDNF production [49].

Although the relationship between exercise and increased BDNF levels is well documented, the underlying biological mechanisms are not entirely clear. Studies in animal models have shown that exercise increases the brain expression of BDNF, particularly in the hippocampus. Among the proposed mechanisms, a key role could be played by fibronectin type III domain-containing protein 5 (FNDC5), a protein expressed not only by muscles during exercise, but also in the brain, as shown by the impaired neuronal development observed in *FNDC5* gene knockouts [50,51]. Interestingly, Wrann et al. showed that exposure of mice to resistance exercise for 30 days induced not only a significant increase in FNDC5 expression in the quadriceps muscle and hippocampus, but also in hippocampal BDNF levels, suggesting a correlation between exercise-induced hippocampal neurotrophin expression and FNDC5 expression. Although the underlying mechanism is still unknown, exercise-induced activation of the peroxisome proliferator-activated receptor gamma coactivator 1-alpha (PGC-1α)/FNDC5/BDNF pathway in the hippocampus has been suggested to be potentially responsible for the positive regulation exerted by FNDC5 on BDNF expression [52].

β-hydroxybutyrate (DBHB), a ketone body whose levels are frequently increased during caloric restriction and after prolonged exercise, was also proposed by Sleiman et al. as a positive regulator of *BDNF* gene expression [53]. Specifically, DBHB treatment of cortical neuron cultures and hippocampal slices from mice undergoing voluntary wheel running was seen to induce an increase in BDNF transcription, compared to that of sedentary mice. According to the authors, this increase could depend on the involvement of histone deacetylases (HDACs), a class of enzymes responsible for epigenetic modifications that determine the regulation of gene transcription. Indeed, HDAC2 and HDAC3 are known to inhibit *BDNF* gene expression, and treatment of primary neurons with DBHB reduces the binding of HDAC2 and HDAC3 to BDNF promoters, thereby promoting their transcriptional activation [53].

More recently, El Hayek et al. identified lactate as an endogenous metabolite, produced during exercise, that can cross the blood-brain barrier and promote learning and memory in a BDNF-dependent manner [54]. Specifically, the authors observed that male mice subjected to 30 days of a running wheel showed higher hippocampal BDNF expression than control mice, along with higher lactate levels, and that treatment of the mice with intraperitoneal injections of a lactate transporter inhibitor abolished the expression of the neuronal BDNF promoter. Interestingly, the involvement of sirtuin 1 (SIRT1), a class III HDAC whose activity is dependent on the levels of high-energy molecules, was proposed in the activation of the PGC-1α/FNDC5 pathway and BDNF expression, since repression of SIRT1 expression by short non-coding RNA (shRNA) abolished lactate-mediated BDNF expression in primary neurons [54].

Overall, these studies support the hypothesis that training is a potent inducer of synaptic plasticity, as the production of particular metabolites during exercise can modulate the activity of enzymes responsible for epigenetic modifications and activate the PGC-1α/FNDC5 pathway, which is crucial for BDNF expression.

#### 3.2.2. NGF

NGF is a growth, survival, and maturation factor of sympathetic and sensory neurons involved in inflammatory hyperalgesia in adulthood. Indeed, its expression is known to increase in skeletal muscle after ischemia and nerve injury, promoting sensitization of sensory nociceptors [55]. In the central nervous system, NGF plays a neuroprotective role through binding to tropomyosin receptor kinase A (TrkA) and p75 neurotrophin receptor (p75NTR) [56]. This interaction promotes the activation of downstream pathways involved in cell survival and differentiation, such as the phosphatidylinositol 3-kinase (PI3K)/protein kinase B (Akt) signaling pathway. In addition, the activation of phospholipase C-γ (PLCγ) by NGF induces intracellular calcium release and subsequent activation of specific calcium-dependent proteins, ion channels, and transcription factors, all of which are crucial events in neuroplasticity [56].

Recently, Wu et al. experimentally induced NGF overexpression in a hippocampal neuronal cell line by taurine administration, observing an increase in TrkA and Akt phosphorylation and, consequently, a significant reduction in apoptosis. The same NGF expression changes were also observed after taurine administration in rats, in association with an improvement in learning and memory, suggesting taurine as a potential candidate against cognitive impairment through inhibition of hippocampal neuronal apoptosis [57].

Although several studies have confirmed the involvement of NGF in mnemonic processes, its role in brain plasticity after physical exercise has not yet been well determined [58]. In this regard, Chae et al. studied the effect of moderate-intensity treadmill exercise on NGF levels and the PI3K/Akt signaling pathway in the hippocampus of aged rats, showing that training suppressed apoptosis by inducing Akt expression and increasing NGF levels [59]. In agreement, Hong et al. observed increased NGF expression and neuronal survival in the dentate gyrus of the hippocampus of rats subjected to 8 weeks of treadmill exercise, confirming the role of neurotrophins in brain health [60].

Human studies have also found an increase in NGF levels in trained subjects. For example, Bonini et al. compared the concentration of circulating NGF in pre-Olympic athletes who were subjected to intense and prolonged exercise with that of healthy individuals, finding significantly higher values in the former group [61]. Moreover, Cho et al. found a significant increase in resting concentrations of BDNF and NGF in obese Korean women who underwent three 40-min sessions of aerobic treadmill exercise per week for 8 weeks, compared with a group of untrained women [62]. Interestingly, the exercise-induced increase in NGF was reported to be intensity-dependent, as the same authors observed in a group of 15 men undergoing treadmill running an increase in NGF of 12%, 19%, and 23%, respectively, following low-, medium- and high-intensity exercise [63].

Although a large body of evidence suggests that exercise is responsible for the upregulation of NGF and subsequent improvement in cognitive function, the underlying mechanism is not yet fully understood, highlighting the need for further studies to understand how this neurotrophin may contribute to the beneficial effects of exercise on brain health.

#### 3.2.3. GDNF

GDNF is a 134 amino acid protein originally identified for its ability to promote dopamine uptake in midbrain dopaminergic neurons, ensuring their survival and differentiation [64]. This neurotrophic factor performs its function through binding to the rearranged during transfection (RET) receptor tyrosine kinase, in association with the GDNF family receptor-α (GFRα). In particular, GDNF is known to initially bind to a GFRα co-receptor and form a high-affinity complex that promotes the trans-phosphorylation of specific tyrosine residues of the homodimeric RET receptor, initiating PI3K, extracellular signal-regulated kinase (Erk), and Akt signaling, which are involved in cell survival [64]. Notably, GDNF has also been identified as the most potent neurotrophic factor for motor neuron survival that can support and maintain the neuromuscular system, both during development and in adulthood, promoting neuroplasticity [65].

Several studies have reported that exercise promotes GDNF expression, although the molecular mechanism responsible for this effect is unclear. In 2013, McCullough et al. evaluated the effect of two weeks of swimming or running exercise on the protein content of GDNF in the spinal cord of young and old rats, finding a significant increase in neurotrophins in the L1-L3 tract of the spinal cords of trained rats compared to sedentary rats. In addition, trained animals showed an increase in the size of motor neurons and the number of GDNF-containing vesicles, highlighting the effectiveness of short-term exercise on GDNF expression [66]. Subsequently, Gyorkos and Spitsbergen hypothesized that high-intensity exercise is effective in altering the protein content of GDNF and in inducing neuromuscular junction plasticity (NMJ) [67]. The authors divided a total of 30 rats into groups—two sedentary groups, two involuntary running groups, one at low and one at high intensity, and two voluntary running groups, one with and one without resistance —and assessed the expression and localization of GDNF in the slow twitch soleus muscle and the fast twitch plantar muscle. The protein content of GDNF was increased by 174% and 161% in the plantar muscle tissue of animals undergoing voluntary resistance training and voluntary non-resistance training, respectively. For soleus muscle, there was a 145% increase in GDNF levels in tissue from animals undergoing low-intensity involuntary training, and a 272% increase in muscle from animals undergoing voluntary non-resistance training. Thus, different exercise protocols can result in significant changes in GDNF expression in different types of muscle tissue [67].

More recently, Peake et al. assessed changes in the expression of cytokines and neurotrophins in a group of nine men subjected to lower body resistance exercise. Interestingly, analysis of biopsies taken from the exercised leg both before exercise and after 2, 24, and 48 h showed an increase in the inflammatory infiltrate, mainly represented by neutrophils and macrophages, together with a significant increase in NGF and GDNF levels, confirming that the expression of these neurotrophins increases as a result of exercise [68].

Finally, an interesting feature of GDNF is its ability to perform retrograde transport in motor neurons. In fact, this neurotrophin released from skeletal muscle can reach first an axon terminal and then, following internalization, the cell body of motor neurons by retrograde axonal transport, promoting their survival [69,70]. Thus, GDNF is responsible for the survival of motor units and ensures the preservation of nerve and muscle function, protecting the neuromuscular system.

#### 3.2.4. NT-3 and NT-4

NT-3 is a protein of 257 amino acids, the expression of which begins during embryonic development and gradually decreases in the postnatal period. In the adult brain, the expression of NT-3 is confined to the dentate gyrus of the hippocampus, where it promotes synaptic plasticity and enhances learning and memory [71]. In addition, NT-3 plays an important role in the survival and function of sensory neurons, as well as in synaptic transmission and maturation of the neuromuscular junction [72]. Exercise-induced NT-3 expression was observed by Koo et al., who assessed cognitive and motor recovery in a group of rats with cortical injury induced by head trauma after exposure to wheel running exercise for three weeks. Noteworthy, the reduction in NT-3 expression due to cortical injury was restored in the trained animals, which also showed an improvement in motor function and cognitive gain, suggesting a role for exercise-induced NT-3 in healing traumatic brain injury [73]. In agreement, Hou et al. evaluated the effect of voluntary movement on NT-3 expression in neurologically impaired rats undergoing a cerebral ischemia-reperfusion procedure by middle cerebral artery occlusion. Surprisingly, a significant reduction in neurological deficits and a significant increase in NT-3 expression were found in trained animals compared to sedentary ones [74].

Regenerative action has also been proposed for NT-4 by Chung et al., who observed a reduction in its expression in the ischemic hemisphere of rats subjected to middle cerebral artery occlusion. Interestingly, treadmill exercise increased the bilateral expression of both the monomeric and dimeric forms of NT-4, as well as its receptor tyrosine kinase B (TrkB), confirming the neuroprotective effect of exercise in improving brain function [75]. More recently, Domínguez-Sanchéz et al. showed the results of a randomized clinical trial of 51 obese men divided into a sedentary group, a high-intensity exercise group, a resistance training group, and a combined high-intensity and resistance exercise group. Analysis of blood samples showed a significant increase in NT-3 and NT-4 levels in subjects undergoing resistance and combined exercise, while higher but not significant values were observed in subjects undergoing high-intensity training [76].

Together, this evidence supports the influence of physical exercise on the expression of NT-3 and NT-4, two important neurotrophins involved in neurogenesis, synaptic transmission and plasticity, and cognitive recovery following brain injury. However, the mechanisms by which exercise increases the expression of these neurotrophins still need important investigation.

## 4. Physical Exercise as a Non-Pharmacological Strategy to Prevent Neurodegeneration

**Figure 2:**
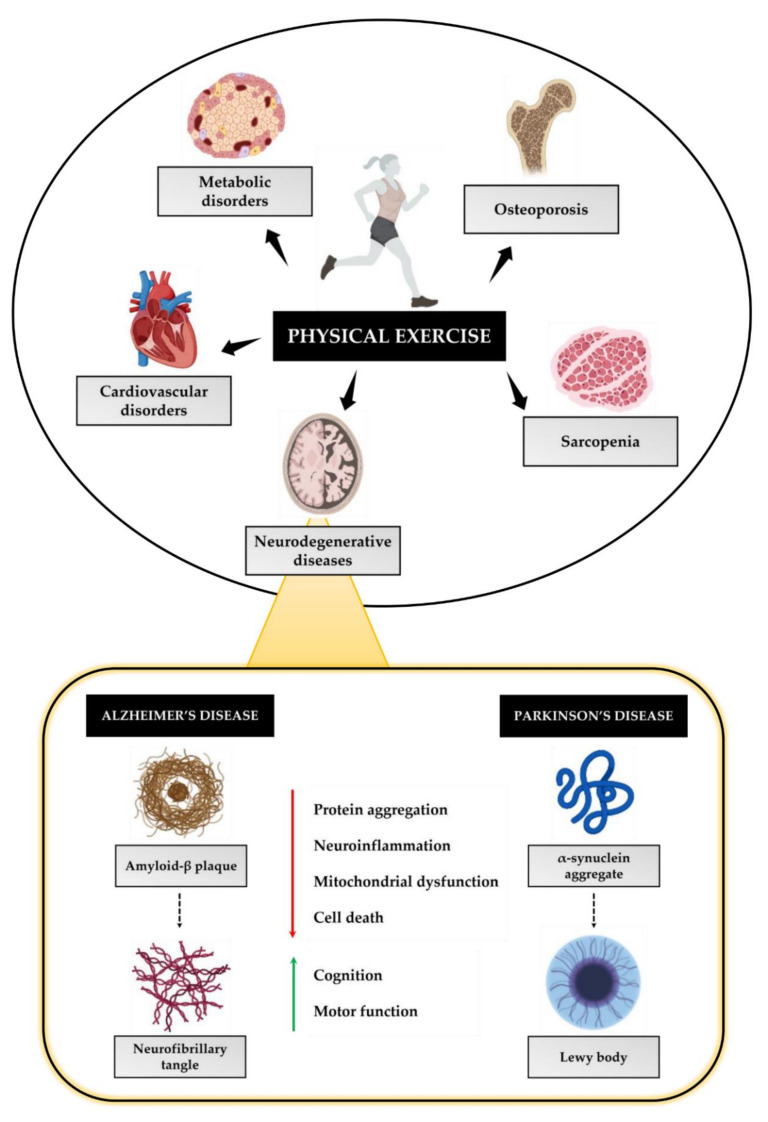
**Beneficial effects of physical exercise.** Physical exercise has a protective effect on a wide variety of disease states, such as osteoporosis, sarcopenia, metabolic disorders, and cardiovascular disorders. Physical exercise is also useful in counteracting the onset and progression of neurodegenerative diseases, including Alzheimer’s disease and Parkinson’s disease, by reducing the formation of neurotoxic protein aggregates, such as β-amyloid protein plaques and lewy bodies. As a result, the protective action of physical exercise involves reducing neuroinflammation, mitochondrial dysfunction, and cell death, and improving cognitive and motor functions.

Physical exercise is generally indicated as the main non-pharmacological therapeutic strategy for a wide variety of diseases, as shown in Figure 2. Indeed, regular exercise can prevent and/or delay the onset of age-related diseases, such as osteoporosis and sarcopenia, as well as metabolic and cardiovascular disorders [15,77]. In addition, numerous scientific studies suggest that physical exercise can counteract the progression of a group of nervous system disorders caused by amyloid deposits, known as neurodegenerative diseases [78]. Although these diseases differ in their pathogenesis, symptoms and the area of the brain initially affected, they share some interesting features, such as neurotoxicity and the presence of insoluble plaques made up of aggregated protein material. Indeed, a misfolding event has been proposed as the underlying mechanism that involves the assumption by a specific protein of an incorrect conformation. This initial event causes the misfolded proteins to aggregate into increasingly large species with a high neurotoxic potential, leading to progressive neuronal loss in certain brain areas [79].

Physical exercise, also through the release of neurotrophins, is an effective strategy for preventing these pathologies, as it helps to preserve memory, neuroplasticity, and neurogenesis.

### 4.1. Physical Exercise and AD

AD is a neurodegenerative disorder characterized by progressive memory loss and inexorable cognitive decline caused by the accumulation of neurotoxic aggregates of the Aβ protein and the phosphorylated protein Tau [80]. This disease is the most common cause of dementia and affects around 30 million people worldwide [81]. Furthermore, recent estimates have reported that, by 2050, the prevalence of the disease will double in Europe and triple worldwide, highlighting the need to use all available tools to counter the onset and progression of this disease [80].

Physical exercise has been suggested as a valuable treatment tool for preclinical and advanced AD, as well as an effective prevention strategy, probably due to the ability of physical exercise to improve cerebral blood flow, increase hippocampal volume, and stimulate neurogenesis [82].

Recently, Hwang et al. evaluated the effect of regular moderate-intensity exercise on improving cognitive function in a mouse model of AD [83]. Specifically, four groups of mice were included: a control group; a group expressing human wild type presenilin-2 (PS-2), a protein that when mutated determines the onset of AD; a group expressing mutated human PS-2; and a group expressing mutated human PS-2 but subjected to treadmill exercise 50 min a day, 5 days a week, for a total of 6 weeks. Interestingly, the group of transgenic animals subjected to exercise showed a greater aptitude for exploring new objects than the untrained transgenic mice. Furthermore, transcriptomic analysis showed an increased expression of factors associated with apoptotic death in the untrained mouse model of AD, suggesting that regular exercise may reverse cellular abnormalities caused by Aβ deposition [83].

Similar results have been observed in human studies in which elderly people diagnosed with AD underwent regular exercise to assess effects on cognition. For example, Morris et al. evaluated the effect of aerobic training on the health of 76 individuals with early AD randomized into two groups, one receiving aerobic exercise of 150 min per week for 26 weeks and a control group receiving stretching exercises [84]. Aerobic exercise promoted not only a modest gain in functional capacity, but also changes in cardiorespiratory fitness positively correlated with improved memory performance and an increase in bilateral hippocampal volume, suggesting the protective role of aerobic exercise against damage induced in the early stages of AD [84].

Although the mechanism by which exercise may counteract neurodegeneration has not yet been fully elucidated, some neurotrophins produced during exercise appear to play a crucial role in mediating this effect. For example, BDNF overexpression is known to counteract AD memory loss in mouse models and non-human primates [85]. Indeed, Aβ aggregates are responsible for reducing BDNF expression by downregulating the cyclic adenosine monophosphate (cAMP) response element binding protein (CREB), a key transcription factor for the expression of genes involved in synaptic plasticity and long-term memory [86]. CREB activity is regulated by multiple kinase pathways, including PI3K/Akt, protein kinase A (PKA), protein kinase C (PKC), which promote CREB activation through phosphorylation, and glycogen synthase kinase 3 beta (GSK3β), which inactivates it. Aβ-induced toxicity reduces CREB activity, either through inactivation of PKA or activation of GSK3β, preventing the expression of genes involved in synaptic plasticity and BDNF expression. This results in low BDNF in the hippocampus and cortex, brain areas involved in higher cognitive functions, impairing memory and learning [87].

A further characteristic of AD is the loss of phenotype and function of basal forebrain cholinergic neurons (BFCNs), whose trophic support is strictly dependent on NGF. An alteration of NGF expression could be responsible for AD-induced BFCN atrophy. Indeed, impaired conversion of the NGF precursor (proNGF) to the mature form (mNGF) was frequently observed in AD brains, together with increased degradation of mNGF and subsequent BFCN atrophy [88]. According to this “cholinergic hypothesis”, the dysfunction of acetylcholine-containing neurons in the basal forebrain contributes significantly to the cognitive decline typical of AD, suggesting a potential therapeutic role of NGF in preserving the phenotype of these neurons [89]. However, the inability of NGF to cross the blood-brain barrier, as well as the adverse effects induced by intraventricular administration, pose difficulties regarding the use of this neurotrophin in AD treatment [90]. Interestingly, intranasal administration of NGF in mouse models of the disease would appear to favor the non-amylogenic pathway of amyloid precursor protein (APP) cleavage, thus reducing Aβ formation [91].

A possible neuroprotective role has also been suggested for GDNF, whose serum levels have been found to be significantly lower in AD patients [92]. Indeed, GDNF overexpression by lentiviral vectors in mouse models of AD was found to be effective in preserving memory and learning, while control animals showed significant cognitive loss. Notably, the effect of GDNF upregulation was correlated with potent BDNF overexpression, suggesting a synergistic action for these neurotrophins in counteracting AD-induced neurodegeneration [93].

Recent scientific evidence also indicates to a neuroprotective role for NT-3 in AD progression, including the study by Yan et al., in which NT-3 overexpression in AD rats was associated with improved cognitive function [94]. Specifically, transplantation of bone marrow-derived mesenchymal stem cells (BMSCs) overexpressing NT-3 was shown to promote neurorigeneration and cognitive gain in AD rats, pointing to β-catenin as a key candidate responsible for this effect. β-catenin is an important member of the Wnt/β-catenin signaling pathway and is mainly present in the cytoplasm. However, translocation of β-catenin from the cytoplasm to the nucleus results in activation of downstream targets that ultimately promote cell survival and proliferation. The authors demonstrated that NT-3 can influence β-catenin expression, although the underlying mechanism remains unclear [94].

Finally, Liu et al. observed the effect of NT-4 overexpression in the hippocampus of AD rats, assessing changes in learning and memory. Specifically, grafting fibroblasts modified with the *NT-4* gene into the hippocampus of AD rats induced a significant survival of cholinergic neurons in the host hippocampus and an equally significant preservation of learning and memory functions [95].

Overall, the evidence reported suggests physical exercise as a surprising and effective non-pharmacological strategy to counteract the cognitive decline that characterizes AD, pointing to neurotrophins produced during exercise as key mediators in preserving higher cognitive function and counteracting disease progression.

### 4.2. Physical Exercise and PD

PD is a degenerative neurological disorder characterized by the aggregation of the protein α-synuclein (α-syn) in the form of lewy bodies, leading to the loss of dopaminergic neurons in the substantia nigra [96,97]. It is the second most prevalent neurodegenerative disease, with an annual incidence in high-income countries of 14 per 100,000 in the total population and 160 per 100,000 in the over-65 population [98].

Despite the significant impact of PD on the elderly population, physical exercise is currently the only valid primary prevention strategy able to reduce the risk of occurrence of the disease [98]. In this regard, Jang et al. evaluated the efficacy of resistance exercise in a mouse model of PD experimentally induced by chronic injection of the neurotoxin 1-methyl-4-phenyl-1,2,3,6-tetrahydropyridin (MPTP) [99]. A total of 30 mice were divided into a sedentary control group, an MPTP-treated group, and an MPTP-treated but resistance-exercise group. At the end of the experimental period, a significant decline in motor coordination was observed in the MPTP-treated mice, whereas the MPTP-treated and resistance-exercise animals showed motor function comparable to that of the control group. Interestingly, significantly lower levels of α-syn were found in mice receiving resistance exercise, along with reduced expression of toll-like receptor 2 (TLR2) and nuclear transcription factor-κB (NF-kB), highlighting the ability of exercise to counteract both the accumulation of neurotoxic aggregates and neuroinflammation and subsequent cell death [99].

The efficacy of resistance exercise has also been observed in human clinical trials, such as that of Schenkman et al., who enrolled a total of 128 subjects with PD, divided into a high-intensity treadmill exercise group, a moderate-intensity exercise group, and a control group. Interestingly, only the high-intensity exercise group reached the non-futility threshold, as fewer motor changes were observed than in the usual care group, providing evidence of the effectiveness of high-intensity exercise in treating PD patients [100].

BDNF and TrkB are known to be widely expressed in the dopaminergic neurons of the substantia nigra, where they participate in the maturation and maintenance of neurons. However, BDNF/TrkB expression is significantly impaired in PD patients, suggesting the involvement of this neurotrophic factor in the PD pathogenesis [101]. Indeed, pathogenic mutations in α-syn, which promote its aggregation and neurotoxicity, are associated with a loss of BDNF and TrkB expression [102]. Furthermore, retrograde axonal transport of BDNF/TrkB, which is essential for the growth and dendritic development of cortical neurons, is impaired in the presence of α-syn fibrillar aggregates, leading to a deficit in BDNF signaling and reduced transcription of specific nuclear targets [103,104]. The reduced expression of BDNF as a molecular signature of PD has led to speculation about its potential role as a therapeutic agent to treat the disease. However, direct administration of exogenous BDNF has not shown significant and lasting improvements in the disease, whereas treadmill exercise has been reported to increase BDNF and GDNF expression in mouse models of PD [105,106]. Thus, physical exercise could exert its neuroprotective action by upregulating various neurotrophins that would act synergistically to reduce the neurodegeneration typical of PD.

NGF also shows an altered expression profile in PD subjects. Indeed, in 2002, Lorigados Pedre et al. detected significantly lower NGF levels in the serum of Parkinsonian rats and individuals compared with control subjects [107]. Subsequently, Wu et al. demonstrated for the first time that LLDT-67, a novel triptolide derivative, has a potent and specific effect on NGF expression in astrocytes in vitro and in vivo, as it protects dopaminergic neurons from MPTP-induced degeneration and stimulates neurotrophin expression, contributing to its neuroprotective effects [108]. More recently, Luo et al. investigated whether the caffeic acid derivative N-propargyl caffeamide (PACA) was able to increase NGF levels against MPTP neurotoxicity in a mouse model of PD [109]. Interestingly, PACA not only enhanced NGF-induced neurite outgrowth, but also improved motor disabilities in diseased mice. Furthermore, PACA was found to increase the conversion of proNGF to active NGF in the midbrain and to sequentially activate the PI3K/Akt, Erk, and CREB signaling pathways, suggesting it as a potent drug candidate for the protection of dopaminergic neurons against neurodegeneration in PD [109].

Regarding GDNF, its ability to promote the survival of nigrostriatal dopaminergic neurons has suggested its possible use as a pharmacological agent in PD treatment. In this regard, several clinical studies have been conducted to assess the efficacy of GDNF administration by intracerebral injections in improving Parkinson’s symptoms or even counteracting disease progression. The efficacy of GDNF was greater when infusion was given into the putamen rather than the ventricles, which bodes well for the use of neurotrophins as a therapy for PD. However, in other clinical trials, treatment with GDNF was unsuccessful, raising doubts about its efficacy in treating PD [110,111]. Nevertheless, the involvement of GDNF in PD has emerged in multiple trials. For example, Gottschalk et al. recently evaluated the efficacy of oral administration of gemfibrozil, a drug approved by the Food and Drug Administration (FDA) for the treatment of hypertriglyceridemia in protecting dopaminergic neurons in a PD animal model. Notably, PD animals treated with gemfibrozil showed improved motor activities in conjunction with increased transcriptional activity of the GDNF gene in astrocytes [112]. Thus, the quality of impaired motor function in PD could be closely related to optimal GDNF expression, although the role of this neurotrophin in maintaining dopaminergic neurons needs further investigation.

Neural stem cell (NSC) transplantation has also been suggested as a promising regenerative medicine therapy for the improvement of Parkinsonian symptoms. In this regard, transplantation of rat neural stem cells endogenously expressing neurotrophin-3 (rNSC-NT3) into Parkinsonian rats treated with 6-hydroxydopamine (6-OHDA) by Gu et al. was shown to improve their spatial learning ability and protect dopamine neurons in the substantia nigra, reversing the main symptoms of PD [113]. Finally, Lingor et al. suggested a synergistic action for NT-4 and GDNF in protecting dopaminergic neurons from damage induced by oxidative stress, one of the main causes of cell death typical of PD, suggesting the use of these neurotrophins for a possible therapeutic application [114].

## 5. Conclusions

Physical exercise is responsible for improving a wide variety of disease states, as well as promoting brain health and preserving cognitive function. Numerous studies have demonstrated the effects of predominantly aerobic exercise on synaptic plasticity, highlighting a significant improvement in neuroplasticity phenomena associated with learning and memory and a gain in motor function. An important role of physical exercise has been observed especially for neurodegenerative diseases, as physical exercise is known to counteract the progression of degenerative disorders affecting the nervous system and to reduce the risk of their onset, preventing the cognitive and physical decline typical of age-related diseases. This neuroprotective effect appears to be achieved by reducing the accumulation of neurotoxic amyloid aggregates and reducing oxidative stress, neuroinflammation, and neuronal death.

Neurotrophins have been suggested as one of the main mediators of this beneficial effect, due to their ability to promote neuronal survival, development, and maintenance, as well as neurogenesis and synaptic plasticity. The expression of neurotrophins is known to be increased by physical exercise through mechanisms that may include the production of specific metabolites and/or the activation of enzymes involved in epigenetic modifications that regulate gene transcription. However, the underlying mechanisms are not yet fully understood, placing limits on the use of neurotrophic factors in the development of potential therapeutic strategies. Notably, neurotrophins could exert their beneficial power on brain health and motor function through a synergistic action, which could be maximized by physical exercise. Indeed, the role of skeletal muscle as an endocrine organ capable of secreting a variety of neurotrophic factors with beneficial and neuroprotective effects is well known. Therefore, it will be necessary to investigate the mechanisms by which neurotrophic factors released not only by the brain, but also by skeletal muscle in response to exercise, could act synergistically to ensure brain health and counteract the cognitive decline of neurodegenerative diseases, including through the development of personalized exercise protocols to achieve maximum neuroprotective action.

## Data Availability

No new data were created or analyzed in this study. Data sharing is not applicable to this article.
